# 

**Published:** 1880-07

**Authors:** 


### JULY, 1880.—CONTENTS.

ORIGINAL COMMUNICATIONS:

The American Horse. By Hon. Geo. B. Loring, M.D., Salem, Mass. .......... 115
Felidæ in Captivity. By Wm. A. Conklin, New York .......... 132
Animal Tuberculosis. By Allen S. Heath, M.D., New York .......... 136
Pathology of Infective and Contagious Diseases. By W. S. Greenfield, M.D., London 142
Colic in Horses. By Geo. H. Berns, D V.S., Brooklyn, N Y. .......... 149
Spaying the Female Canine .......... 153
Cutting and Interfering in Horses. By Daniel E. Dowd, New York .......... 155

EDITORIAL .......... 157

CASE DEPARTMENT.

Rupture of the Gastrocnemius, &c. .......... 163
Selected Cases Hospital Reports .......... 165
Inversion of Uterus in the Cow .......... 166
Four Cases of Fibroma .......... 168

PROGRESS OF VETERINARY SCIENCE.

Wool-Sorters Disease—The Hog Plague—A New Anthelmintic—Contractility of Capillary Blood Vessels—Production of Sex At Will—Sheep Rot—Microscopic Organisms in Ice—Traumatism of the Muscles of the Sub-Lumbar Region—A Celosomian Monstrosity of the Calf—Scabies—Curare—Carnivorous Habits of Bees—The Germ Disease Theory—Growth as a Function of Cells—Regeneration of the Vetreous Humor in Animals—Placenta of Elephas—A New Preservative Fluid—Quinine for Hypodermic Use—Gelatine Suppositories—Kidney Worm—Pamphlet Box .......... 171

MISCELLANY.

Zoological Garden—Infected Cattle—Personal—New State Board of Health—Hog Cholera—Extent of Lung Disease—Inoculation of Rabies—Domestication of Certain Ruminants, &c —A New Theory Concerning Gout .......... 182

ADVERTISERS.

John Reynders & Co., Veterinary Instruments, 303 Fourth Avenue, Second Cover Page; F. G. Otto & Sons, Veterinary Instruments, 64 Chatham St., Page 1; D. C. Newell & Sons, Horse Bedding, Foot W. 19th St, Page 2; R. M. Stivers, Carriages, etc, E. 31st St., J. H. Belding, Veterinary Medical Books, Third Ave., cor. 28d St., Third Cover Page; Columbia Veterinary College, Fourth Cover Page.

